# Deriving BMI-referenced, sex-specific fat mass index cutoff values in older Saudi adults: a cross-sectional secondary analysis

**DOI:** 10.3389/fnut.2026.1900590

**Published:** 2026-07-23

**Authors:** Maram Alsadi, Mahmoud M. A. Abulmeaty, Aljazi Bin Zarah, Adel Alhamdan

**Affiliations:** Community Health Sciences Department, College of Applied Medical Sciences, King Saud University, Riyadh, Saudi Arabia

**Keywords:** body composition, body mass index, fat mass index, obesity, older adults, Saudi Arabia

## Abstract

**Background/objectives:**

Age-related changes in body composition may reduce the ability of body mass index (BMI) to accurately reflect adiposity in older adults. Fat mass index (FMI), which expresses fat mass relative to height squared, may provide a more direct estimate of adiposity. However, BMI-referenced, sex-specific FMI cutoff values have not been well characterized among older Saudi adults. This study aimed to derive sex-specific cutoff values for FMI, body fat percentage (%BF), and fat mass corresponding to selected BMI thresholds among older Saudi adults.

**Methods:**

This cross-sectional secondary analysis included 1,954 adults aged ≥60 years who attended 15 randomly selected primary healthcare centers in Riyadh, Saudi Arabia. Anthropometric and bioelectrical impedance analysis-derived body composition data were analyzed. Receiver operating characteristic (ROC) curve analysis and the Youden index were used to derive cutoff values, and DeLong’s test for correlated ROC curves was used to compare BMI-referenced discriminatory performance.

**Results:**

Across the examined BMI thresholds, FMI demonstrated high discriminatory performance relative to BMI in both men and women. For older men, FMI cutoffs corresponding to BMI > 27 kg/m^2^, BMI ≥ 30 kg/m^2^, and BMI > 31 kg/m^2^ were 7.90, 9.48, and 9.71 kg/m^2^, respectively. The corresponding values in older women were 10.30, 12.10, and 13.00 kg/m^2^. DeLong testing generally supported higher AUCs for FMI than for %BF and fat mass, although the difference between FMI and fat mass at BMI ≥ 25 kg/m^2^ in men was not statistically significant.

**Conclusion:**

This study provides BMI-referenced, sex-specific FMI cutoff values for older Saudi adults. FMI may serve as a complementary body composition index when BIA-derived fat mass is available. Because the cutoffs were derived using BMI as a pragmatic reference framework in a cross-sectional sample from primary healthcare in Riyadh, they should be interpreted as BMI-corresponding values rather than definitive clinical risk thresholds. External validation against clinical outcomes and reference methods for body composition is required before broad clinical implementation.

## 1 Introduction

Obesity is a major global public health concern, with rising prevalence across age groups, including older adults (1). The World Obesity Federation has projected a substantial increase in the number of adults living with obesity by 2035 (2). In the United States, obesity affects more than 40% of adults aged 60 years and older (3). In Saudi Arabia, overweight and obesity are also highly prevalent among adults (4–6), including older adults; recent national data indicate that obesity affects 20.4% of older men and 39.8% of older women (General Authority for Statistics (7)). Excess body weight contributes to several noncommunicable diseases, including diabetes, kidney disease, and cardiovascular disease (8), and high BMI is an important contributor to the noncommunicable disease burden in Saudi Arabia (9).

Body mass index (BMI) remains the most widely used anthropometric measure for classifying overweight and obesity because it is simple, inexpensive, and practical in clinical and epidemiological settings. The World Health Organization defines overweight as BMI ≥ 25 kg/m^2^ and obesity as BMI ≥ 30 kg/m^2^ (10). However, alternative BMI thresholds have also been proposed for older adults. The Nutrition Screening Initiative and Lipschitz proposed BMI > 27 kg/m^2^ as an indicator of unhealthy weight in older adults (11, 12), while a meta-analysis of 32 cohort studies found that BMI > 31 kg/m^2^ was associated with increased all-cause mortality in this age group (13).

Despite its practical value, BMI has important limitations as an indicator of adiposity in older adults. It reflects total body mass relative to height but does not distinguish between fat, lean, and bone mass. This limitation is especially relevant with aging, as older adults commonly experience reductions in lean body mass, increases or redistribution of fat mass, and reductions in resting energy expenditure (14, 15). These age-related changes may weaken the relationship between BMI and adiposity, contributing to misclassification, particularly in individuals with reduced muscle mass and relatively high fat mass. Higher body fat percentage and lower muscle mass have also been associated with frailty, supporting the need to consider body composition rather than body weight alone (16, 17).

Age-related changes in body composition are also closely linked to reductions in resting energy expenditure and basal metabolic rate, which may complicate the interpretation of body weight and adiposity in older adults. In individuals with overweight or obesity, Karagun and Baklaci (18) emphasized the importance of individualized basal metabolic rate assessment, supporting the need to interpret adiposity measures within a broader metabolic context.

Body composition-based indices may complement BMI. Percentage body fat (%BF) reflects the proportion of body weight that is fat, whereas fat mass index (FMI), calculated as fat mass divided by height squared, expresses absolute fat mass relative to stature (19). FMI may therefore provide a more specific estimate of adiposity because it focuses on the fat component of body weight while adjusting for height. This distinction is clinically relevant in older adults, in whom muscle and fat mass may change in opposite directions.

In Saudi Arabia, evidence on body composition cutoffs for older adults remains limited. One study reported mean %BF values of approximately 29% in older men and 40% in older women (20). Other Saudi studies in adults found that mean %BF exceeded commonly used international reference values and that women had higher %BF than men (21, 22). However, those studies either relied on international cutoffs that may not fully reflect Saudi population characteristics or were conducted in general adult populations rather than specifically in older adults (21, 22). Abulmeaty et al. derived fat-index cutoff values in Saudi adult men and found that FMI showed high diagnostic performance for BMI-defined obesity (23), but the study included only younger adult men, so its findings may not be directly applicable to older adults.

This gap is important because the relationship between BMI and body fat indices may vary by age, sex, population, and measurement method. Older adults experience changes in body composition that may alter the interpretation of BMI and fat-related indices (14, 15), and women generally have higher body fat than men at comparable BMI levels (20–22). Therefore, sex-specific body fat index values corresponding to commonly used BMI thresholds are needed for older Saudi adults.

Importantly, BMI is not a gold-standard measure of adiposity. In this study, BMI served as a pragmatic reference framework because it remains the most commonly used clinical and epidemiological classification system for overweight and obesity (11, 12). Accordingly, the derived values should be interpreted as FMI, %BF, and fat mass cutoffs that correspond to established BMI thresholds, rather than as definitive thresholds for predicting clinical outcomes, metabolic risk, or mortality.

This study aimed to derive sex-specific cutoff values for FMI, %BF, and fat mass that correspond to selected BMI thresholds among older Saudi adults. A secondary aim was to compare the BMI-referenced discriminative performance of these body fat indices using Receiver Operating Characteristic (ROC) analysis and formal Area under the Curve (AUC) comparisons.

## 2 Materials and methods

### 2.1 Study design and data source

This study was a cross-sectional secondary analysis of data from a previously completed research project (Project No. 10-med121902), funded by the National Plan for Science, Technology, and Innovation (MAARIFAH) at King Abdulaziz City for Science and Technology in Saudi Arabia. The original project, conducted from January 2015 to April 2017, included older adults aged ≥60 years who attended 15 randomly selected primary healthcare centers in Riyadh City, Saudi Arabia (34).

The original project assessed the internal environment of primary healthcare centers and the health status of older adults, including nutritional status, muscle strength, physical function, anthropometric characteristics, and body composition (34). The present secondary analysis was appropriate for the current objective because the dataset included the variables needed to derive BMI-referenced body fat index cutoffs, including height, weight, fat mass, %BF, and calculated BMI. For this secondary analysis, ethical approval was obtained from the Institutional Review Board (IRB) of the College of Medicine at King Saud University (reference number: 23/0924/IRB; approval date: 2 March 2025).

### 2.2 Study population and eligibility criteria

The original dataset included 2,045 older adults. For the present analysis, participants were excluded if they had cancer, neurological disorders, major musculoskeletal disorders, structural deformities, or had been hospitalized or undergone surgery within the 6 months preceding enrollment. These criteria were applied to reduce the potential influence of acute illness, severe disability, or major disease-related changes in body composition. After applying these criteria, 1,954 older adults were included in the final analysis. In addition, as part of the original body composition measurement protocol, individuals with dehydration, edema, metal implants, or pacemakers were excluded because these conditions may affect the flow of electrical current and compromise the accuracy of BIA-derived estimates.

### 2.3 Anthropometric and body composition measurements

Anthropometric and body composition measurements were collected as part of the original project using standardized field procedures (34). Body weight and height were measured, and BMI was calculated as weight in kilograms divided by height in meters squared.

$\mathrm{BMI}=\text{weight}\;(\mathrm{kg})/{\text{height}}^{2}\;(m^{2})$

Body composition was assessed using a bioelectrical impedance analysis device (BC-418, Tanita Corporation, Tokyo, Japan). This device was used to estimate fat mass and %BF, as described previously for this older-adult dataset (19). FMI was calculated by dividing fat mass in kilograms by height in meters squared, consistent with the established definition of FMI (17):

$\mathrm{FMI}=\mathrm{fat}\;\text{mass}\;(\mathrm{kg})/{\text{height}}^{2}\;(m^{2})$

Bioelectrical impedance analysis was used because it is practical, non-invasive, relatively inexpensive, and feasible for field-based studies involving large samples. Nevertheless, BIA estimates may be influenced by hydration status, body water distribution, recent food intake, and measurement conditions, particularly in older adults (24). Therefore, the resulting cutoff values should be interpreted as BIA-derived estimates and should not be considered directly interchangeable with values obtained using reference methods such as dual-energy X-ray absorptiometry (DXA).

### 2.4 BMI thresholds used as pragmatic reference categories

In this study, BMI was not treated as a gold-standard measure of adiposity. Rather, BMI thresholds were used as pragmatic reference categories because BMI remains widely used in clinical practice, epidemiological surveillance, and public health classification (11–13). The purpose of the analysis was to derive body fat index values corresponding to established BMI thresholds, not to validate FMI against a direct clinical outcome or a reference method for body composition. Accordingly, the ROC-derived cutoff values in this study should be interpreted as BMI-corresponding values rather than thresholds validated against an independent gold-standard measure of adiposity or against clinical outcomes such as metabolic disease, functional decline, or mortality.

Four BMI thresholds were examined:

1. BMI ≥ 25 kg/m^2^, corresponding to the World Health Organization classification for overweight [10].
2. BMI ≥ 30 kg/m^2^, corresponding to the World Health Organization classification for obesity [10].
3. BMI > 27 kg/m^2^, proposed as an indicator of unhealthy weight in older adults by the Nutrition Screening Initiative and Lipschitz (11, 12).
4. BMI > 31 kg/m^2^, reported in a meta-analysis as a BMI level associated with increased all-cause mortality risk in older adults (13).

Using these thresholds allowed the derived fat-index values to be interpreted in relation to commonly used and older-adult-relevant BMI classifications.

### 2.5 Statistical analysis

Data were analyzed using the Statistical Package for the Social Sciences (SPSS), version 22.0 (IBM Corp., Armonk, NY, United States). Categorical variables were summarized as frequencies and percentages. Age was summarized as mean ± standard deviation, whereas BMI, %BF, fat mass, and FMI were summarized as median and interquartile range (IQR) because they were not normally distributed. The Chi-square test was used to compare categorical variables between men and women. Age was compared between men and women using the independent-samples t-test, whereas BMI, %BF, fat mass, and FMI were compared using the Mann–Whitney U test. Spearman’s correlation coefficient was used to assess associations among BMI, fat mass, %BF, and FMI. A two-sided *p*-value <0.05 was considered statistically significant.

Receiver operating characteristic (ROC) curve analysis was used to evaluate the ability of fat mass, %BF, and FMI to discriminate between participants classified above and below each selected BMI threshold. AUC and 95% confidence interval were calculated for each body fat index. The Youden index was used to identify the cutoff value that maximized the combined sensitivity and specificity for each index.

AUCs for FMI, %BF, and fat mass were formally compared using DeLong’s test for correlated ROC curves in MedCalc. Statistical significance was set at *p* < 0.05. Because the reference categories were based on BMI, all ROC and DeLong results were interpreted as reflecting BMI-referenced discriminatory performance rather than evidence of independent clinical or prognostic superiority.

## 3 Results

### 3.1 Characteristics of the study participants

A total of 1,954 older adults aged ≥60 years were included in the final analysis, of whom 1,101 were men, and 853 were women (Table 1). Men were significantly older than women. Marked sex differences were also observed in marital status, educational level, and employment status.

**Table 1:** Characteristics of older adults who participated in the study.

Characteristics	Males (*N* = 1,101)	Females (*N* = 853)	*p*-value
	*N* (%)	*N* (%)	
Sex	1,101 (56.4)	853 (43.6)	
Age, Mean ± SD	66.65 ± 6.53	64.8 ± 6.23	<0.001
Marital status			
Married	1,053 (95.7)	507 (59.4)	
Single	6 (0.5)	10 (1.2)	<0.001
Widow	39 (3.5)	282 (33.1)	
Divorced	3 (0.3)	54 (6.3)	
Education levels			
Illiterate	205 (18.6)	486 (57.0)	
Primary	325 (29.5)	210 (24.6)	<0.001
Intermediate and secondary	412 (37.4)	124 (14.5)	
University or above	159 (14.5)	33 (3.9)	
Occupation			
Employed	258 (23.4)	71 (8.3)	<0.001
Not employed	843 (76.6)	782 (91.7)	
%BF, Median (IQR)	29.8 (8.6)	40.4 (8.1)	<0.001
Fat mass, Median (IQR)	22.9 (11.5)	29.2 (12.7)	<0.001
FMI, Median (IQR)	8.37 (4.2)	12.32 (5.2)	<0.001
BMI, Median (IQR)	28.0 (6.5)	30.5 (7.0)	<0.001
BMI classification			
Underweight	18 (1.6)	7 (0.8)	
Normal	251 (22.9)	102 (12.0)	
Overweight	452 (41.0)	280 (32.8)	<0.001
Obese	380 (34.5)	464 (54.4)	
BMI >27 classification			
≤27	470 (42.7)	216 (25.3)	
>27	631 (57.3)	637 (74.7)	<0.001
BMI >31 classification			
≤31	800 (72.7)	479 (56.2)	<0.001
>31	301 (27.3)	374 (43.8)	

Women had significantly higher BMI, %BF, fat mass, and FMI than men (Table 1). The median BMI was 28.0 kg/m^2^ in men and 30.5 kg/m^2^ in women. Similarly, the median values for %BF, fat mass, and FMI were higher among women than men, indicating substantial sex-related differences in body composition.

BMI classification also differed significantly by sex (Table 1). According to the WHO BMI classification, overweight was more common among men, whereas obesity was more common among women. Using the older-adult unhealthy-weight threshold of BMI > 27 kg/m^2^, 57.3% of men and 74.7% of women were classified above this threshold. Using the BMI threshold associated with increased mortality risk in older adults, BMI > 31 kg/m^2^, the corresponding proportions were 27.3% in men and 43.8% in women.

Data are presented as numbers and percentages, mean ± standard deviation, or median (interquartile range), as appropriate. The Chi-square test was used for categorical variables. Age was compared between men and women using an independent-samples t-test. BMI, %BF, fat mass, and FMI were compared using the Mann–Whitney U test. BMI, body mass index; %BF, percentage body fat; FMI, fat mass index; IQR, interquartile range. + BMI classification based on the World Health Organization criteria: underweight, normal weight, overweight, and obesity. ++ BMI > 27 kg/m^2^ was used as the threshold for unhealthy weight in older adults. +++ BMI > 31 kg/m^2^ was used as a threshold previously associated with increased all-cause mortality risk in older adults.

### 3.2 Correlations among BMI and body fat indices

BMI, %BF, fat mass, and FMI were strongly and significantly correlated in both men and women (Table 2). In men, BMI showed strong positive correlations with %BF, fat mass, and FMI. Similar patterns were observed in women. Among the body fat indices, FMI was highly correlated with fat mass and %BF in both groups.

**Table 2:** Correlation coefficients among body composition variables in older men.

Variables		BMI	%BF	Fat mass	FMI
Older men	BMI	1.000	0.810**	0.923**	0.938**
	%BF	0.810**	1.000	0.925**	0.957**
	Fat mass	0.923**	0.925**	1.000	0.974**
	FMI	0.938**	0.957**	0.974**	1.000
Older women	BMI	1.000	0.839**	0.915**	0.963**
	%BF	0.839**	1.000	0.921**	0.949**
	Fat mass	0.915**	0.921**	1.000	0.958**
	FMI	0.963**	0.949**	0.958**	1.000

BMI, Body mass index (kg/m^2^); FMI, Fat mass index (kg/m^2^); %BF, percentage body fat. **Spearman correlation is significant at the 0.01 level.

These findings indicate strong associations among BMI and body composition indicators in this sample. However, because these indices reflect different aspects of body size and adiposity, high correlations should not be interpreted as evidence that they are interchangeable.

### 3.3 ROC analysis and derived cutoff values for body fat indices

**Figure 1:**
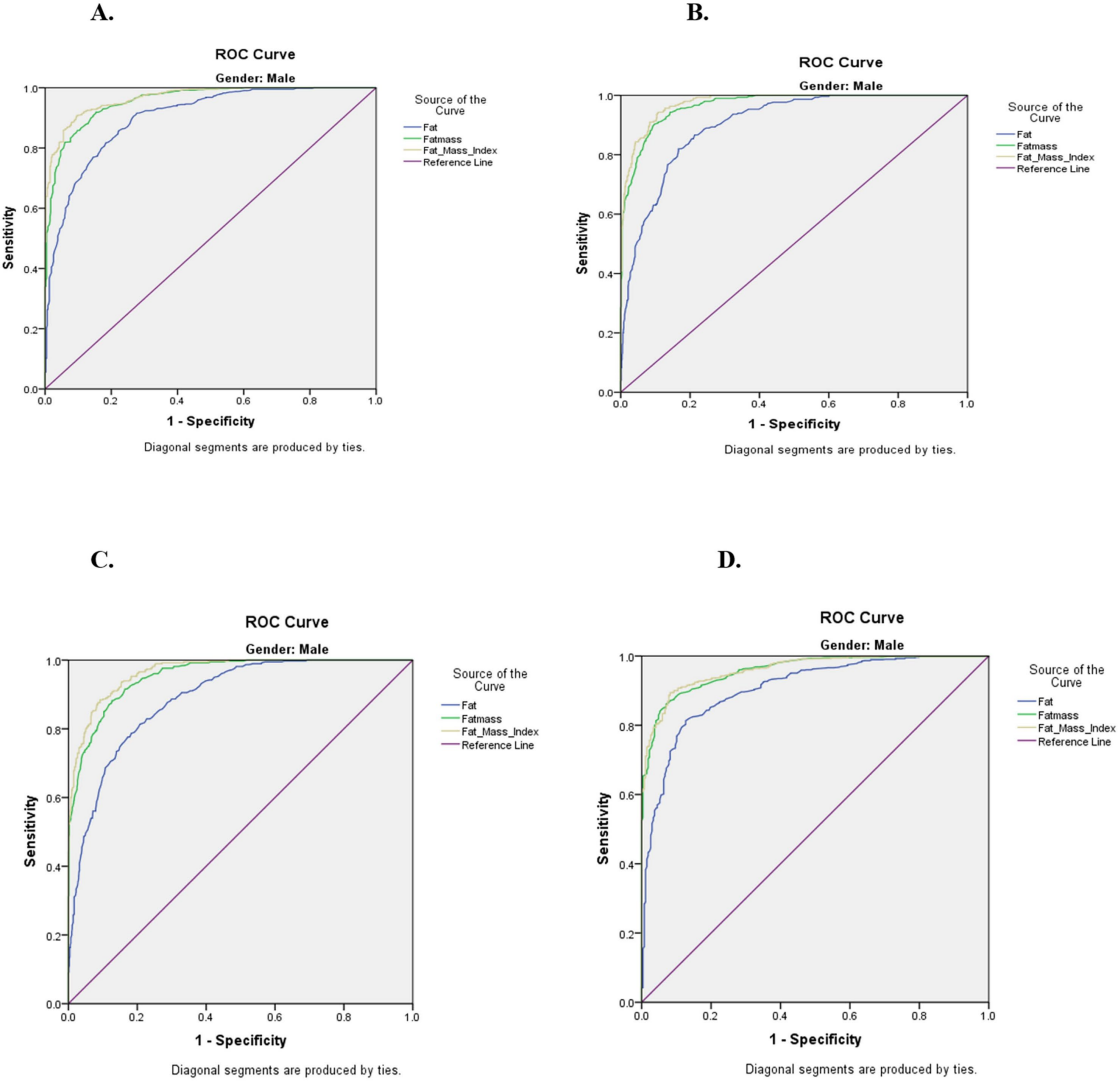
ROC analysis for %BF, fat mass, and FMI based on BMI classifications in older men: **(A)** BMI > 27 kg/m^2^, **(B)** BMI > 31 kg/m^2^, **(C)** BMI ≥ 30 kg/m^2^, and **(D)** BMI ≥ 25 kg/m^2^.

**Figure 2:**
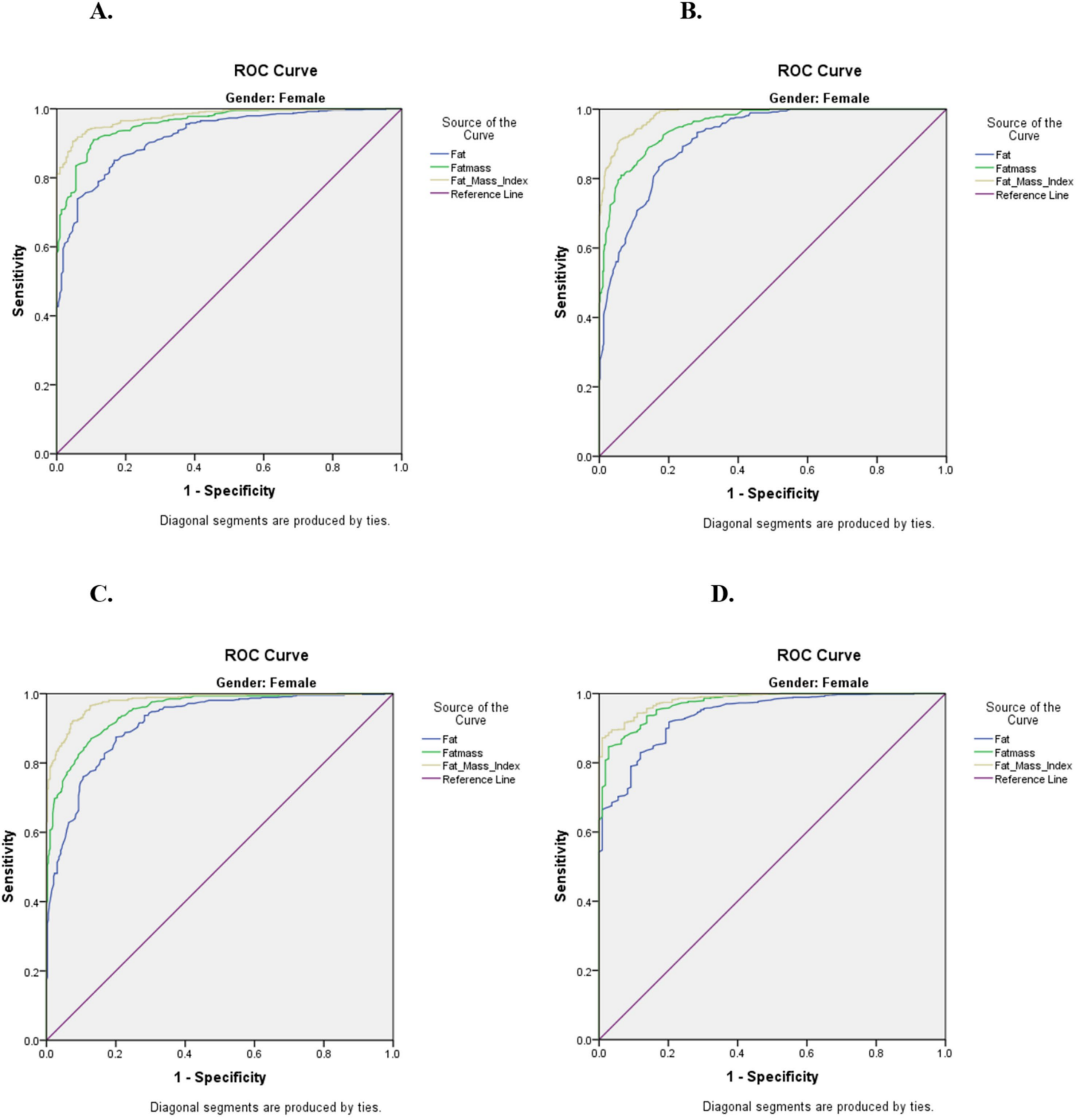
ROC analysis for %BF, fat mass, and FMI based on BMI classifications in older women: **(A)** BMI > 27 kg/m^2^, **(B)** BMI > 31 kg/m^2^, **(C)** BMI ≥ 30 kg/m^2^, and **(D)** BMI ≥ 25 kg/m^2^.

Table 3 presents the AUC values, Youden indices, and derived cut-off points for %BF, fat mass, and FMI across the selected BMI thresholds in older men and women, respectively. The corresponding ROC curves are shown in Figures 1, 2. Across the examined BMI thresholds, FMI had the highest AUCs, followed by fat mass and %BF. DeLong’s test was used to assess whether these AUC differences were statistically significant (Supplementary Tables S1 and S2).

**Table 3:** ROC analysis and optimal cut-off points for body fat indicators across different BMI thresholds in older men.

Gender	BMI	Measure	AUC	95% CI	Youden index	Cut-off
Older men	BMI ≥ 25	%BF	0.906	0.887–0.923	0.69	27.3
		Fat mass	0.959	0.945–0.970	0.79	19.1
		FMI	0.961	0.948–0.972	0.81	6.81
	BMI > 27	%BF	0.902	0.883–0.919	0.64	27.4
		Fat mass	0.957	0.943–0.968	0.77	21.0
		FMI	0.967	0.955–0.977	0.80	7.90
	BMI ≥ 30	%BF	0.889	0.870–0.908	0.61	31.1
		Fat mass	0.955	0.941–0.966	0.75	25.2
		FMI	0.966	0.954–0.976	0.79	9.48
	BMI > 31	%BF	0.902	0.883–0.919	0.65	31.5
		Fat mass	0.967	0.955–0.977	0.81	26.8
		FMI	0.976	0.965–0.984	0.83	9.71
Older women	BMI ≥ 25	%BF	0.938	0.919–0.953	0.7162	34.0
		Fat mass	0.972	0.958–0.982	0.8193	23.8
		FMI	0.982	0.970–0.990	0.8589	9.71
	BMI > 27	%BF	0.921	0.901–0.938	0.6842	37.5
		Fat mass	0.960	0.944–0.972	0.8040	24.7
		FMI	0.977	0.964–0.986	0.8517	10.30
	BMI ≥ 30	%BF	0.915	0.894–0.933	0.6745	39.3
		Fat mass	0.951	0.935–0.965	0.7422	28.2
		FMI	0.978	0.966–0.987	0.8451	12.10
	BMI > 31	%BF	0.914	0.894–0.932	0.6630	41.0
		Fat mass	0.955	0.938–0.968	0.7484	29.9
		FMI	0.984	0.973–0.991	0.8492	13.00

BMI, body mass index; FMI, fat mass index; %BF, percentage body fat; AUC, area under the curve; CI, confidence interval. Cutoff units are percentages for %BF, kg for fat mass, and kg/m^2^ for FMI. BMI ≥25 kg/m^2^ corresponds to the World Health Organization overweight classification [10]; BMI ≥30 kg/m^2^ corresponds to the World Health Organization obesity classification; BMI >27 kg/m^2^ corresponds to the older-adult unhealthy weight threshold; BMI >31 kg/m^2^ corresponds to the BMI level associated with increased mortality risk in older adults.

Among older men, using BMI > 27 kg/m^2^ as the threshold for unhealthy weight, the derived cutoff values were 27.4% for %BF, 21.0 kg for fat mass, and 7.90 kg/m^2^ for FMI (Table 3; Figure 1). For BMI ≥ 30 kg/m^2^, corresponding to WHO-defined obesity, the derived cutoff values were 31.1% for %BF, 25.2 kg for fat mass, and 9.48 kg/m^2^ for FMI. For BMI > 31 kg/m^2^, the threshold previously associated with increased all-cause mortality risk in older adults, the derived cutoff values were 31.5% for %BF, 26.8 kg for fat mass, and 9.71 kg/m^2^ for FMI. For BMI ≥ 25 kg/m^2^, corresponding to WHO-defined overweight, the derived cutoff values were 27.3% for %BF, 19.1 kg for fat mass, and 6.81 kg/m^2^ for FMI.

Among older women, the derived cutoff values were 37.5% for %BF, 24.7 kg for fat mass, and 10.30 kg/m^2^ for FMI at BMI > 27 kg/m^2^ (Table 3; Figure 2). For BMI ≥ 30 kg/m^2^, the derived cutoff values were 39.3% for %BF, 28.2 kg for fat mass, and 12.10 kg/m^2^ for FMI. For BMI > 31 kg/m^2^, the derived cutoff values were 41.0% for %BF, 29.9 kg for fat mass, and 13.00 kg/m^2^ for FMI. For BMI ≥ 25 kg/m^2^, the derived cutoff values were 34.0% for %BF, 23.8 kg for fat mass, and 9.71 kg/m^2^ for FMI.

Overall, FMI yielded the highest BMI-referenced AUCs across sex-specific analyses and BMI thresholds (Table 3). DeLong testing showed that FMI had significantly higher AUCs than %BF at all examined thresholds in both sexes (Supplementary Tables S1 and S2).

FMI also had significantly higher AUCs than fat mass in most comparisons; the exception was the BMI ≥ 25 kg/m^2^ threshold in men, where the difference between FMI and fat mass was not statistically significant (Tables S1 and S2). These results support the potential use of FMI as a complementary index for interpreting adiposity when BIA-derived fat mass is available.

## 4 Discussion

The present study derived sex-specific cutoff values for FMI, %BF, and fat mass that correspond to commonly used BMI thresholds for older adults. FMI showed consistently high BMI-referenced AUCs across all examined thresholds in both men and women. These findings suggest that FMI may be a useful complementary index for interpreting adiposity when BIA-derived body composition data are available. However, because BMI was used as a pragmatic reference rather than a gold-standard measure of adiposity, the derived values should be interpreted as BMI-corresponding cutoffs rather than definitive diagnostic, prognostic, or clinical risk thresholds.

The need for sex-specific body fat index values is supported by the marked sex differences observed in this sample. Older women had significantly higher BMI, %BF, fat mass, and FMI than older men, and obesity-related BMI categories were more prevalent among older women. These findings align with previous Saudi data in adults showing higher body fat levels among women than among men (20–22). These differences are clinically relevant because the same BMI value may correspond to different levels of adiposity in men and women. Sex-specific FMI values may therefore provide more meaningful interpretation than a single cutoff applied to all older adults. However, because men in the present sample were significantly older than women, the observed sex-specific cutoff values may partly reflect differences in age distribution in addition to biological sex differences in adiposity. Although the absolute age difference was modest (66.65 vs. 64.80 years), this issue should be considered when interpreting the sex-specific estimates.

FMI showed higher BMI-referenced AUC values than %BF and fat mass across the BMI thresholds examined. DeLong’s test showed significantly higher AUCs for FMI than for %BF in all sex-specific analyses and for FMI than for fat mass in most analyses. The only exception was the BMI ≥ 25 kg/m^2^ threshold in men, where FMI and fat mass had very similar AUC values. This pattern is biologically plausible because FMI expresses fat mass relative to height squared, thereby adjusting for body size while focusing specifically on the fat component of body weight (17). Nevertheless, these findings should be interpreted as evidence of stronger BMI-referenced discrimination, not as proof that FMI predicts clinical outcomes better than other indices.

The findings are broadly consistent with those of Abulmeaty et al. (21), who reported high diagnostic performance of FMI for BMI-defined obesity among Saudi adult men. However, the FMI cutoff for WHO-defined obesity in the present study was higher in older than in younger Saudi men. This difference may reflect age-related changes in body composition, including reductions in lean mass and shifts in fat distribution (14, 15). The present study extends earlier Saudi evidence by including older adults of both sexes and by deriving values across several BMI thresholds relevant to older populations.

Comparisons with international studies should be interpreted cautiously because body fat cutoff values vary by population characteristics, age group, reference criterion, and body composition assessment method. Alemán-Mateo et al. (25) reported FMI cutoffs for obesity in older Latin American adults that varied by the BMI criterion used. Di Renzo et al. (26) reported a higher %BF at a BMI of approximately 27 kg/m^2^ in older Italian women than that observed in the present study. Studies using metabolic syndrome or metabolic conditions as reference outcomes have also reported cutoff values that differ from BMI-corresponding cutoffs (31, 32). These differences emphasize that fat-index cutoff values are not interchangeable across populations, assessment methods, or clinical purposes.

The relatively high adiposity levels observed in this Saudi older-adult sample may reflect several population-specific factors, including rapid lifestyle and nutritional transitions, urbanization, changes in dietary patterns, and lower physical activity levels. Cultural and social factors may also contribute to sex-specific obesity patterns, particularly among older women, who showed higher BMI, %BF, fat mass, and FMI in the present study (27). These regional characteristics may partly explain why BMI-corresponding body fat index values in older Saudi adults differ from those reported in Italian, Latin American, and other international cohorts.

A key methodological issue is the use of BMI as the reference framework. BMI was used because it remains widely applied in clinical practice, epidemiological surveillance, and public health classification (11–13)). However, BMI is not a direct measure of adiposity and cannot distinguish fat mass from lean mass. Therefore, the derived FMI, %BF, and fat mass values should not be interpreted as validated against an independent gold standard. Rather, they report body fat index values corresponding to established BMI categories in this sample. This distinction is essential to avoid overestimating the clinical meaning of the findings.

Another important consideration is the use of BIA to assess body composition. BIA is practical, non-invasive, and feasible for large field-based studies, particularly in primary healthcare settings. However, BIA estimates may be affected by hydration status, recent food intake, body water distribution, and measurement conditions, factors that are especially relevant in older adults (33). BIA-derived values may also differ from those obtained using DXA or other reference methods (28, 29). Therefore, the proposed cutoff values should be considered specific to BIA-derived estimates and should not be assumed interchangeable with DXA-derived cutoff values.

The clinical relevance of the proposed FMI cutoffs lies in their potential use as complementary interpretive values when body composition assessment is available. In primary healthcare or geriatric nutrition settings, FMI may help clinicians determine whether an older adult’s fat mass aligns with commonly used BMI-defined categories. This may be useful when BMI alone is difficult to interpret, particularly in individuals with reduced muscle mass, altered body composition, or suspected sarcopenic obesity. Nevertheless, FMI reflects only the fat component of body weight and does not provide direct information on lean mass, muscle strength, physical performance, or fat distribution. This is particularly important in older adults, in whom excess adiposity may coexist with reduced muscle mass and impaired function, commonly referred to as sarcopenic obesity. Therefore, FMI should not be used in isolation to characterize obesity-related risk in geriatric populations. Future studies should evaluate composite approaches that combine adiposity indicators with muscle-related measures, including appendicular skeletal muscle mass, handgrip strength, gait speed, and other functional indicators. However, FMI should not replace a comprehensive assessment. In older adults, adiposity should ideally be interpreted alongside waist circumference, muscle strength, functional status, comorbidities, dietary intake, and metabolic indicators.

The interpretation of FMI may also be strengthened when considered alongside metabolic indicators, including resting energy expenditure or basal metabolic rate. Because aging is associated with changes in lean mass, fat mass, and energy expenditure, FMI alone cannot capture the metabolic heterogeneity of older adults with overweight or obesity (30). Future studies should examine whether combining FMI with metabolic measures improves risk stratification beyond BMI or adiposity-based classification alone.

This study has several strengths. It included a relatively large sample of older Saudi adults, allowing sex-specific analyses. It also examined multiple BMI thresholds, including WHO thresholds and older-adult-relevant thresholds, thereby providing a broader interpretive framework. In addition, ROC curve analysis, the Youden index, and DeLong testing enabled systematic derivation and comparison of cutoff values for multiple body fat indices.

The study also has limitations. First, its cross-sectional design precludes causal inference or prediction of future clinical outcomes. Second, BMI was used as a pragmatic reference rather than a gold-standard measure of adiposity; therefore, the derived cutoffs should be interpreted as BMI-corresponding values rather than definitive indicators of health risk. Third, body composition was assessed using BIA, which is sensitive to hydration status and other measurement conditions. Although individuals with dehydration, edema, metal implants, and pacemakers were excluded according to the original BIA measurement protocol, detailed information on other conditions that may influence hydration status or fluid distribution, such as chronic kidney disease or heavy diuretic use, was not fully available in this secondary dataset. Therefore, some residual measurement error related to hydration or fluid balance cannot be excluded. Fourth, participants were recruited from primary healthcare centers in Riyadh city, which may limit generalizability to older adults in other regions of Saudi Arabia, rural communities, or institutionalized populations. In addition, men and women differed significantly in age, although the absolute difference in mean age was modest. Because body composition changes with advancing age, the derived sex-specific cutoffs may have been partly influenced by age distribution. Future studies should examine age-adjusted and age-stratified FMI cutoffs in independent cohorts of older adults. Finally, the proposed cutoff values were not externally validated in an independent cohort. External validation using independent Saudi samples, reference body composition methods, and clinically relevant outcomes is needed before these cutoffs can be recommended for broad clinical implementation.

## 5 Conclusion

This study derived BMI-referenced, sex-specific cutoff values for FMI, %BF, and fat mass corresponding to commonly used BMI thresholds in older Saudi adults. FMI showed consistently high BMI-referenced AUCs across sex-specific analyses and BMI categories, suggesting that it may serve as a complementary body composition index when BIA-derived fat mass is available, particularly when BMI interpretation is ambiguous.

Because these cutoff values were derived using BMI as a pragmatic reference rather than a gold-standard measure of adiposity, they should be interpreted as BMI-corresponding values rather than definitive diagnostic, prognostic, metabolic, or mortality-risk thresholds. FMI should therefore be used as part of a broader clinical and geriatric assessment, rather than as a standalone diagnostic criterion.

Future studies should externally validate these cutoff values in independent Saudi cohorts and compare them with reference body composition methods and clinically relevant outcomes, including metabolic syndrome, cardiometabolic risk, functional status, sarcopenic obesity, and mortality. Such validation is needed to determine whether FMI cutoffs provide additional clinical value beyond BMI-based classification.

## Data Availability

The raw data supporting the conclusions of this article will be made available by the authors, without undue reservation.
