# Supplementary material for: Deriving BMI-referenced, sex-specific fat mass index cutoff values in older Saudi adults: a cross-sectional secondary analysis
Source: Front Nutr. 2026 Jul 23;13:1900590. doi: 10.3389/fnut.2026.1900590 (PMC13441698; doi:10.3389/fnut.2026.1900590)
Supplement: Supplementary file 1 [file Table_1.docx]

Supplementary Table S1. DeLong’s test results comparing AUC values among body fat indices in older men.

| **BMI threshold** | **Comparison** | **AUC 1** | **AUC 2** | **Difference** | **DeLong p-value** |
| --- | --- | --- | --- | --- | --- |
| BMI ≥25 kg/m² | FMI vs %BF | 0.961 | 0.906 | 0.055 | <0.001 |
| BMI ≥25 kg/m² | FMI vs fat mass | 0.961 | 0.959 | 0.002 | 0.343 |
| BMI ≥25 kg/m² | Fat mass vs %BF | 0.959 | 0.906 | 0.053 | <0.001 |
| BMI >27 kg/m² | FMI vs %BF | 0.967 | 0.902 | 0.065 | <0.001 |
| BMI >27 kg/m² | FMI vs fat mass | 0.967 | 0.957 | 0.010 | <0.001 |
| BMI >27 kg/m² | Fat mass vs %BF | 0.957 | 0.902 | 0.055 | <0.001 |
| BMI ≥30 kg/m² | FMI vs %BF | 0.966 | 0.889 | 0.077 | <0.001 |
| BMI ≥30 kg/m² | FMI vs fat mass | 0.966 | 0.955 | 0.012 | <0.001 |
| BMI ≥30 kg/m² | Fat mass vs %BF | 0.955 | 0.889 | 0.065 | <0.001 |
| BMI >31 kg/m² | FMI vs %BF | 0.976 | 0.902 | 0.074 | <0.001 |
| BMI >31 kg/m² | FMI vs fat mass | 0.976 | 0.967 | 0.009 | 0.003 |
| BMI >31 kg/m² | Fat mass vs %BF | 0.967 | 0.902 | 0.065 | <0.001 |

Pairwise comparisons between correlated AUCs were performed using DeLong’s test in MedCalc.

AUC 1 and AUC 2 correspond to the first and second body fat indices listed in each pairwise comparison, respectively. Statistical significance was set at p < 0.05.
